# Posterior Malleolar Fractures: From the "Forgotten Fragment" to Modern Concepts in Management

**DOI:** 10.7759/cureus.94681

**Published:** 2025-10-15

**Authors:** Ahmed Mohamed, Usman Fuad, Alaa Elasad, Sagaurav Shrestha, Aasim Hagroo, Ioannis P Pengas

**Affiliations:** 1 Trauma and Orthopaedics, Royal Cornwall Hospitals NHS Trust, Truro, GBR; 2 General Practice, Zagazig University, Zagazig, EGY

**Keywords:** direct posterior fixation, posterior inferior tibiofibular ligament, posterior malleolus fracture, post-traumatic ankle joint osteoarthritis, syndesmotic instability, trimalleolar fracture

## Abstract

Posterior malleolar fractures (PMFs) affect ankle stability and patient prognosis. The posterior malleolus (PM) provides an articular surface, syndesmotic stability, and tibiotalar joint stability. Computed tomography (CT) scanning has changed the practice from size-based management to understanding the bigger picture, which includes considering fragment morphology, articular involvement, and syndesmotic stability. Treatment options range from conservative management in specific populations and fracture patterns to operative management for complex fractures that affect ankle joint integrity. Operative management options are controversial among orthopaedic surgeons. This focused review examines the anatomy, diagnosis, and various management strategies for PMFs.

## Introduction and background

Posterior malleolar fractures (PMFs) are common and occur in up to 44% of ankle fractures; however, they remain one of the most debated injuries in ankle trauma [1]. They are often part of complex bimalleolar or trimalleolar patterns. Treatment for both lateral and medial malleolar fractures is well established; however, there is no consensus on the best treatment for PMFs [2]. The posterior malleolus (PM) was once known as the "forgotten fragment." It is now recognized as a critical determinant of ankle stability. The PM is important because it provides an articular surface for weight bearing, anchors the posterior inferior tibiofibular ligament (PITFL), and prevents the talus from sliding backward. Injury to this fragment can affect both joint stability and long-term function. Undertreatment of PMFs often results in posttraumatic osteoarthritis (PTOA) and affects patients’ function [3-5].

For many years, treatment decisions were based only on the size of the posterior fragment seen on plain radiographs. Fragments larger than 25% of the articular surface were fixed, whereas smaller fragments were ignored or treated with syndesmotic screws [6]. This simple rule has shaped practice for decades, but it is based on very limited evidence published in the 1940s [5,7]. Current evidence has shown that fragment size alone is insufficient to guide management. Factors such as displacement, syndesmotic instability, cartilage impaction, and fracture morphology on computed tomography (CT) scans are now recognized as key considerations in decision-making [8-11]. Recent advances in CT have further improved our understanding of this fracture. Different CT cuts help guide the operative decision, type of fixation, and direction of metalwork insertion. Multiple classification systems have emerged to better characterize injuries beyond simple size measurements [12].

Despite this progress, there is still no universal agreement on the best method for managing PMFs. Surgeons continue to debate whether to fix the fracture directly, rely on indirect reduction, or stabilize only the syndesmosis. Different surgical approaches and fixation methods are available, each with its own advantages and risks. Outcomes are closely linked to the accuracy of reduction, but achieving this in practice can be difficult, especially in comminuted or osteoporotic fractures [1,13-15].

Owing to these controversies, PMFs remain a challenging topic. Understanding the anatomy, biomechanics, and current treatment options is essential to improve patient care. This article reviews the recent literature, summarizes the different surgical strategies, and highlights ongoing debates and future directions in the management of PMFs.

## Review

Anatomy and stabilizing ligaments

The PM represents the posterior distal tibial articular surface. Biomechanically, the PM is important for ankle stability and function. It has three functions. First, it forms approximately 30% of the tibial plafond articular surface, allowing load distribution across the tibiotalar joint. Second, it maintains syndesmotic stability through the PITFL attachment. Third, it forms a posterior buttress that prevents posterior translation of the talus during ankle motion. Loss of posterior malleolar support has been shown to decrease the tibial plafond contact area by up to 35%, increase the peak contact pressure by nearly 40%, and permit posterior talar subluxation. These changes become particularly significant when the fracture fragment involves more than 25% of the articular surface [16-18].

**Figure 1:**
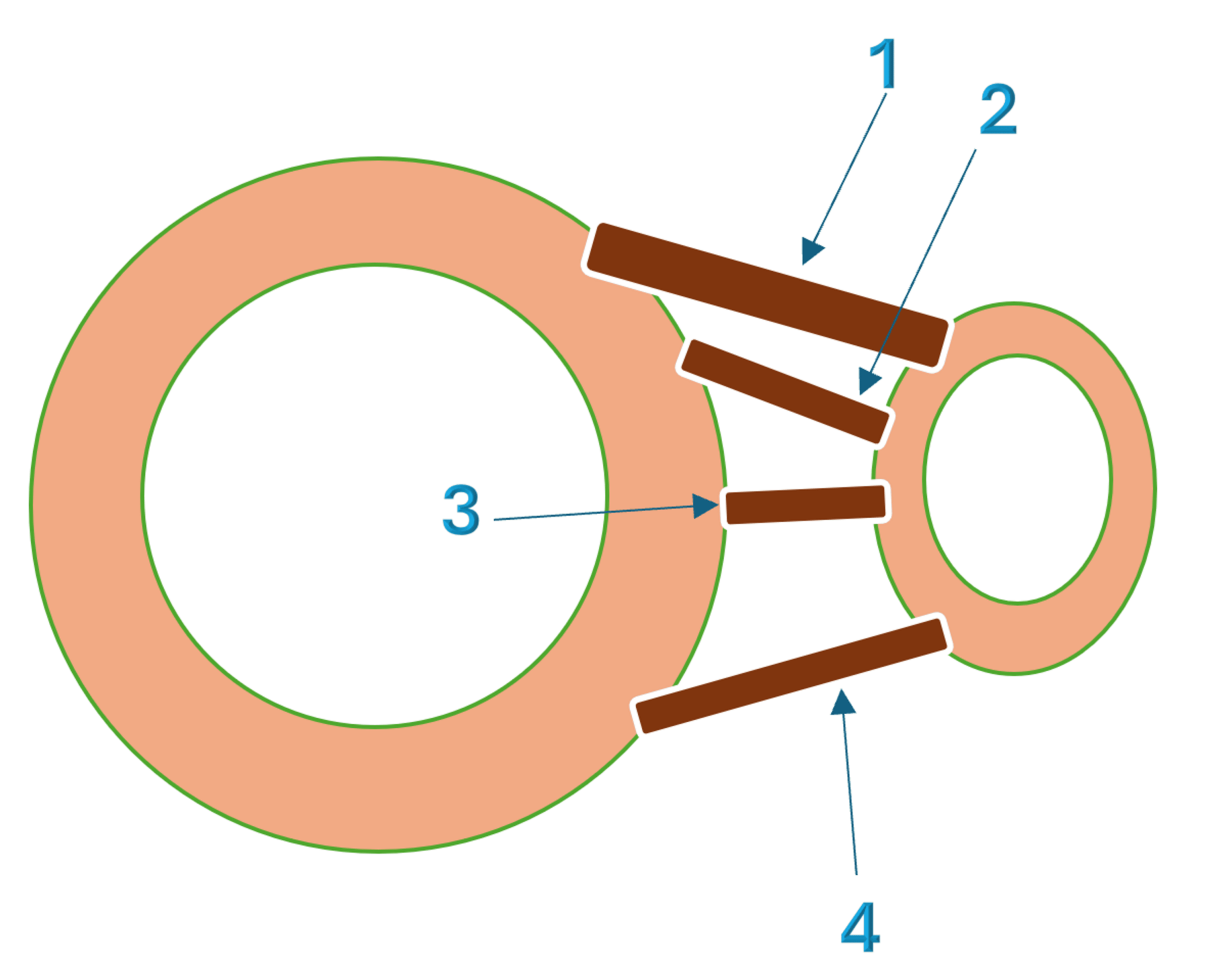
Anatomy of the syndesmosis Image Credit: Alaa Elasad This figure has been created using the Infinite Painter application and annotated using Microsoft Office 365 (Microsoft® Corp., Redmond, WA, USA). 1 - Superficial fibers of the posterior inferior tibiofibular ligament; 2 - Deep fibers of the posterior inferior tibiofibular ligament; 3 - Interosseous ligament; 4 - Anterior inferior tibiofibular ligament.

The PITFL is a key stabilizer of the distal tibiofibular syndesmosis and is involved in the pathoanatomy of PMFs. Studies have shown that the PITFL provides 42% of syndesmotic stability. Inadequate surgical treatment of PMFs leads to failure of joint biomechanics and syndesmotic instability, eventually leading to PTOA [19]. The PITFL is composed of superficial and deep fibers (Figure 1). The superficial fibers extend from the posterior tibial tubercle to the posterior distal fibula. Deep, or transverse, fibers arise from the posterior tibial plafond and attach to the posterior margin of the fibular articular surface. The deep fibers act as a buttress against talar translation. In the setting of a PMF, the PITFL remains firmly attached to the avulsed bony fragment. This explains the mechanism of syndesmotic failure without a true syndesmosis lesion [20]. From this perspective, theories have been proposed to explain why fixation of the PM can restore syndesmotic stability without the need for separate syndesmotic screw fixation [21].

Mechanism of injury

PMFs primarily result from rotational and axial loading mechanisms. This mechanism occurs in the Lauge-Hansen classification’s supination-external rotation (40%-50%) and pronation-external rotation (60%-70%) patterns. The external rotation component creates tension in the PITFL, facilitating posterior fragment avulsion. Axial loading causes articular impaction [22].

The foot position at the time of injury determines the fragment morphology. Plantarflexion produces smaller fragments, as the wider anterior talus engages the tibia. Dorsiflexion results in larger fragments, as the narrower posterior talus allows deeper penetration into the posterior tibia. The rotational direction influences the fracture orientation: external rotation creates posterolateral fragments (Haraguchi Type I, 67%), whereas combined mechanisms produce medial extension patterns (Type II, 19%) or small avulsion fragments (Type III, 14%) [7]. High-energy mechanisms produce comminution and impaction, whereas low-energy injuries in osteoporotic patients create larger fragments with less displacement. Associated injuries, including one- or two-malleolar fractures and syndesmotic disruption, occur frequently [23-25].

Classification

**Figure 2:**
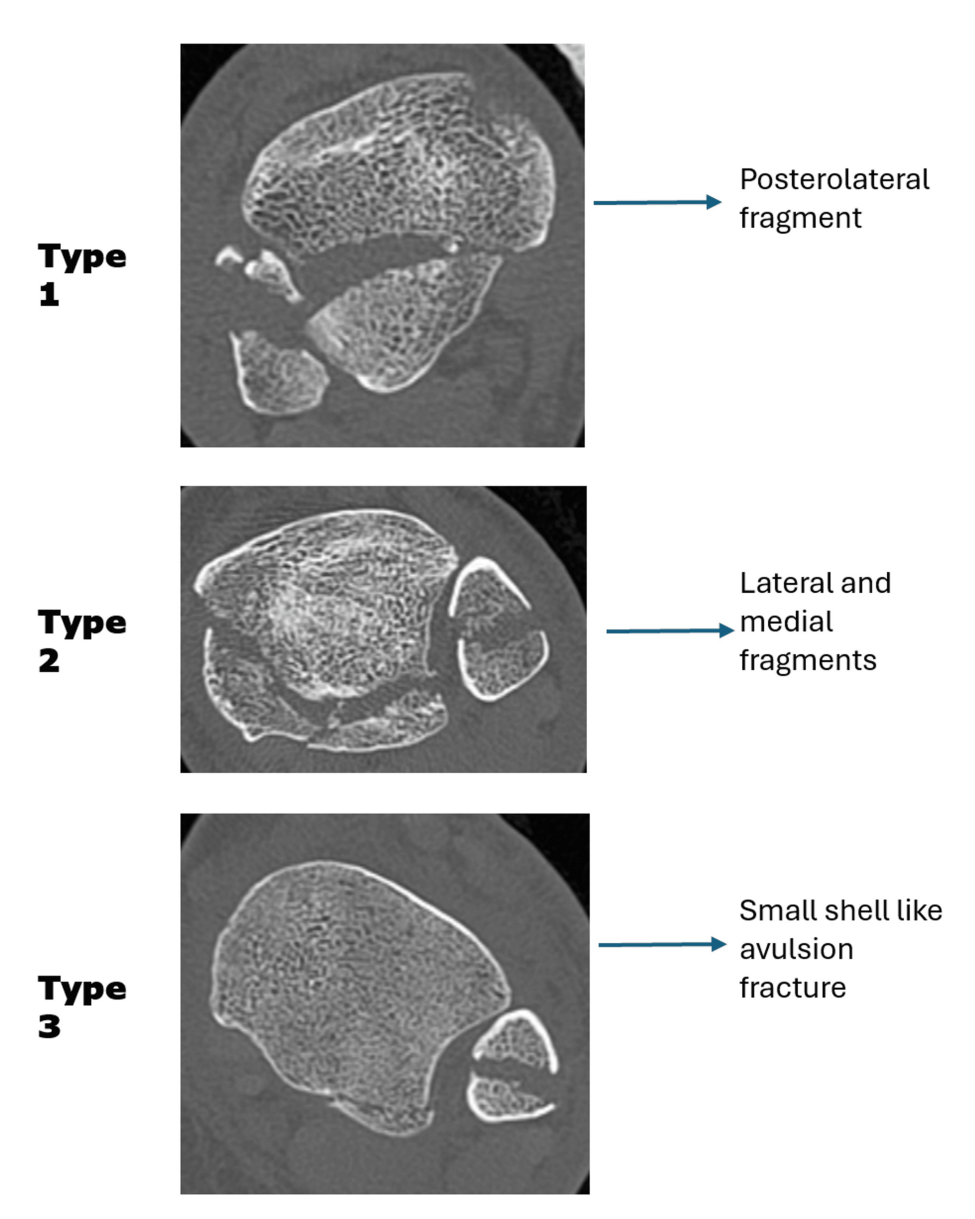
Haraguchi classification of posterior malleolus fractures Image Credit: Ahmed Mohamed This figure has been created using Microsoft Office 365 (Microsoft® Corp., Redmond, WA, USA). The subimages in this figure are genuine from patients in my hospital; no consent was taken, as there is no patient-identifying information in the images.

The Haraguchi classification is one of the earliest CT-based systems and remains the most commonly used classification system (Figure 2). PMFs are classified into three main patterns. Type 1 is the posterolateral oblique fragment, which is the most frequent and usually amenable to fixation through a posterolateral approach. Type 2 fractures extend more medially, often involving a larger portion of the tibial plafond and sometimes requiring both posterolateral and posteromedial exposures. Type 3 is a small shell-like avulsion at the posterior lip, often associated with PITFL avulsion. The advantage of Haraguchi’s system is its simplicity. It is easy to apply in practice and helps surgeons quickly categorize injury patterns from axial CT cuts [23].

The Bartoníček classification is more detailed and was designed to guide surgical decision-making. It distinguishes five types: small extra-incisural fragments (Type 1), posterolateral fragments (Type 2), posteromedial fragments (Type 3), large fragments involving both columns (Type 4), and fractures in which almost the entire posterior plafond is separated (Type 5). This system provides more information for preoperative planning by considering not only the fragment size and location, but also how the fracture impacts joint stability and syndesmotic integrity [26]. The Bartoníček classification, with proposed management options, is presented in Table 1.

**Table 1:** Bartoníček classification with proposed management options ORIF: open reduction internal fixation

Type	Description	Surgical Implication/Management
Type 1	Small extra-incisural fragment	Often stable, conservative management may be enough in most cases.
Type 2	Posterolateral fragment	Needs ORIF through the posterolateral approach.
Type 3	Posteromedial fragment	Needs ORIF through the posteromedial approach.
Type 4	Large fragment involving both the posterolateral and the posteromedial columns	Often needs combined posterolateral and posteromedial approaches, or an extensile posterior approach.
Type 5	Nearly the entire posterior plafond separated	Most severe injury; Needs complex reconstruction; Often requires posterior plating and possible bone grafting if comminuted.

Clinical assessment

Physical examination revealed typical ankle fracture findings, with maximal tenderness over the PM. Careful assessment includes evaluation of blisters, particularly posterolaterally over the approach landmark. Clinical documentation is crucial for all ankle fractures. This should include careful assessment and documentation of past medical history - in particular, diabetes, peripheral neuropathy, peripheral vascular disease, renal impairment, osteoporosis, baseline mobility status, smoking, and alcohol intake. Additionally, skin integrity and distal neurovascular status are very important to document, especially in ankle fracture-dislocations [27]. 

Imaging

Plain radiography is the first diagnostic investigation. It underestimates fragment size because of the overlapping bony structures and the oblique orientation of the PM. The lateral view best demonstrates PM fragments. The anteroposterior (AP) and mortise views may show associated injuries, such as widening of the medial clear space, or absence of tibiofibular overlap, which indicate ligamentous instability [28].

CT scanning is essential for surgical planning. It is now standard practice to perform CT scanning in cases of PM involvement, regardless of size on plain radiographs. CT provides three-dimensional (3D) views that allow for an accurate assessment of fracture size, site, direction, comminution, impaction, articular involvement, intercalary fragments (ICFs), and syndesmotic disruption [12,29]. This information guides surgeons in deciding the best approaches and metalwork needed for each patient individually [30].

Management strategies

Conservative Management

Conservative treatment remains an option in carefully selected cases of isolated PMFs. A fracture is considered stable when the talus remains centered under the tibia with no subluxation, the mortise is symmetric on plain films, and stress views or CT scans show no syndesmotic widening or medial clear space increase. Indications include fragments with less than 2 mm of articular step-off, small extra-articular shell fragments such as Haraguchi Type III, and cases with no syndesmotic instability. It can also be considered in elderly, low-demand or non-ambulatory patients, or in patients with severe comorbidities where surgery is not safe [14]. Treatment usually involves a below-knee cast or functional boot for approximately six weeks. During this period, serial radiographic follow-up is necessary to detect any early failure. Weight-bearing views should be taken at 7-10 days and again at two to three weeks to confirm that the mortise remains stable. Long-term outcomes are good in carefully selected cases. If talar shift, mortise widening, or loss of reduction occurs, surgical fixation is required to restore ankle congruity and prevent long-term arthritis [31-33].

Surgical management

Approach Selection

Surgical management of PMFs requires careful preoperative planning, which begins with CT-based decision-making. Patient positioning is crucial for the smoothness of the operation. Patients are usually positioned either prone or in a lazy lateral position. In trimalleolar fractures, surgeons often fix the PM first, as fixing the fibula initially may obstruct the surgical field and make controlling the posterior fragment more difficult. The posterolateral approach is the most commonly used approach. The incision runs between the peroneal and Achilles tendons, with the sural nerve and short saphenous vein being the main structures at risk. Its advantage is that it provides good visualization of posterolateral fragments and allows a double window for the simultaneous fixation of associated fibular fractures [34].

A posterior paratendinous approach (just lateral to the Achilles tendon) has been described as a modification of the posterolateral approach. It provides a single, central window with less dissection and is useful when there is a central posterior fragment without lateral or fibular involvement. However, it is less versatile when the fibula requires fixation [4].

The posteromedial approach is preferred when the fracture extends medially. This incision runs along the posterior border of the tibia, just medial to the Achilles tendon, providing direct access to the posteromedial fragment. The posterior tibial artery, vein, and nerve are at high risk when this approach is used [14].

For multifragmentary fractures, or those extending across both columns, an extensile posterior approach can be used to address both the posterolateral and posteromedial fragments through a single incision. This carries the advantage of direct access but at the cost of greater soft tissue dissection, a higher risk of wound complications, and potential injury to the tibial neurovascular bundle [13].

Surgical Planning and Techniques

Fixing the PM based on the fragment size only in the initial lateral radiographic views is a historic approach. A decision was made to perform open reduction internal fixation (ORIF) in cases of more than 25% involvement of the articular surface. This approach originated from a study published in 1940 that reviewed only eight ankle fracture cases with PM involvement, making it a weak basis for modern practice [35]. In cases of less than 25% involvement, indirect fixation with syndesmotic stabilization was offered to stabilize the ankle mortise [36]. This outdated approach is now being challenged, with current evidence suggesting that factors such as syndesmotic instability, intra-articular step-off greater than 1-2 mm, plafond impaction, and the presence of ICFs should be considered, rather than size alone. Recent studies have shown great controversy regarding the best way to fix PMF. It depends mainly on the surgeon’s experience and technique. We will discuss these options, along with supporting evidence [37,38].

In general, PMFs can be fixed directly or indirectly. Indirect reduction involves two common mechanisms. The first is through AP lag screws. It relies on the instant reduction of the PM once the lateral malleolar fracture has been reduced and fixed, as the lateral and posterior malleoli are attached to the PITFL. AP lag screws are placed under radiographic guidance. This method is not commonly used in current practice and is not recommended by most surgeons. A recent study by Liu and Huang proved that the anterior-to-posterior screw technique is associated with fewer functional outcomes than the posterior-to-anterior technique [39]. Limitations include the inability to address articular impaction, difficulty in achieving anatomic reduction in comminuted fractures, and a significant risk of malreduction when the fragment is rotated. Additionally, it is biomechanically illogical to fix the tibia to the broken fragment.

The second indirect reduction technique is the stabilization of the distal tibiofibular syndesmosis using screws or suture-button devices, without addressing the fragment itself. Several comparative studies have shown that syndesmotic screw fixation can provide functional outcomes equivalent to direct reduction and fixation stabilization, with a high rate of patient satisfaction. These findings suggest that, in selected cases (particularly when the posterior fragment is small, minimally displaced, or indirectly reduced), syndesmotic fixation alone remains a reasonable treatment option, although this remains an area of ongoing debate [40-45].

Arthroscopic-assisted reduction can fix some of the problems associated with indirect techniques. It allows direct visualization of the articular surface, while percutaneous reduction and fixation are performed under fluoroscopic guidance. This technique facilitates the elevation of impacted fragments, assessment of joint congruity, and removal of loose bodies. Studies have shown comparable outcomes to open approaches, with the potential advantage of fewer wound complications. However, it requires specific skills and a high level of expertise [46].

Direct reduction includes ORIF of fractures. ORIF allows direct visualization of the fragment and anatomical fixation. Fixation can be performed using screws, screws with washers, or plates and screws. Several plate options are available, including one-third tubular plates and locking distal tibial plates [47]. A recent study by Chang et al. confirmed that plates provide more rigidity and stability than screws under axial loading and Achilles forces [48]. Additionally, surgeons can address syndesmotic disruption or fix the syndesmosis using anchors in the case of small avulsion fractures [5,49]. Several studies have shown that direct fixation provides a more reliable anatomic reduction and restores syndesmotic stability better than indirect methods. A systematic review confirmed that ORIF of the fragment leads to superior patient outcomes and less malreduction compared to leaving it unfixed or using only indirect techniques [50]. Clinical series using the posterolateral approach also reported high rates of anatomic reduction and good functional scores, with no clear increase in complications [51,52]. Biomechanical and clinical studies have highlighted that direct fixation provides superior syndesmotic stability compared with trans-syndesmotic screws or suture-button devices, and this can often remove the need for additional syndesmotic fixation if stability is restored [53,54].

Other Considerations

Even after direct fixation of the PM, associated syndesmotic instability can persist in a few cases due to concomitant injury to the AITFL or interosseous membrane; therefore, syndesmotic testing by hook test, external rotation stress test, or cotton test, under image intensifier guidance, is still necessary even after direct ORIF. In up to one-third of cases, patients may still demonstrate residual diastasis, requiring additional trans-syndesmotic fixation. This underscores the importance of assessing the entire syndesmotic complex, rather than assuming that stability is fully restored by posterior fixation alone [53-55].

The ICF represents a challenge in PMFs and has gained increasing recognition for its clinical significance. The ICF is defined as a free osteochondral fragment located between the main PMF and the remaining intact tibial plafond. Recent studies have demonstrated that ICFs are present in approximately 30%-40% of PMFs, with a higher prevalence in Bartoníček-Rammelt Type 3 fractures. Studies have suggested that the ICF should be removed if it is <2 mm, and fixed if it is >2 mm. Biomechanically, the ICF acts as a destabilizing element that can prevent anatomic reduction if not properly addressed, leading to articular incongruity and potential PTOA. Current surgical techniques emphasize the importance of identifying ICFs preoperatively using CT imaging, and addressing them through direct visualization via posterior surgical approaches. Failure to recognize and adequately reduce the ICF has been associated with persistent articular step-offs greater than 2 mm, and inferior functional outcomes. Therefore, the presence of an ICF should be considered an indication for operative intervention, regardless of the size of the main PM fragment, as indirect reduction techniques are generally insufficient to achieve anatomic restoration of the articular surface when an ICF is present [38,56-60].

Outcomes and complications

The strongest predictor of long-term outcomes is the achievement of an anatomic articular reduction. In trimalleolar patterns, a persistent postoperative step-off of the PM > 1 mm increases the chance of PTOA nearly fourfold and is linked to worse functional scores at mid- to long-term follow-up [61]. Radiographic PTOA is noticed in 25%-34% of ankle fractures involving the PM. Meticulous reduction appears to decrease this risk [62]. Direct posterior fixation improves reduction quality compared with percutaneous or indirect techniques, particularly for multifragmentary fragments or displacement ≥5 mm, without a clear increase in overall complication rates [63].

In a large study of 300 surgically treated PMFs, the overall in-hospital complication rate was 13.7%. Delayed wound healing occurred in approximately 5% of patients, infection requiring revision in approximately 2%, reoperation in approximately 7%, and secondary ankle fusion in approximately 2% of patients at a mean nine-year follow-up. The posterolateral approach did not increase wound problems compared with the lateral approach when performed by experienced surgeons [64]. Hardware symptoms remain common. Registry-level data show implant removal rates of approximately 12%-27% after ankle ORIF (not specific to PM fixation), making this an important point for patient counseling [65].

Finally, prospective cohort work highlights that, even with radiographic union, patients may report persistent stiffness and activity limitations at 6-12 months, underlining the importance of anatomical reduction and planning an early rehabilitation program. Early physiotherapy and encouraging patients to bear weight early are recommended to decrease stiffness and facilitate an early return to activities [66,67].

Limitations and future directions

Although we now have better imaging, surgical approaches, and fixation techniques, there are still clear gaps in the evidence for PMF management. Most studies are retrospective and include mixed groups of patients, with different definitions of fragment size, fracture type, and outcomes. Randomized controlled trials are rare in this field. Many studies also have small sample sizes and short follow-ups, which limit our understanding of long-term problems such as PTOA or the need for ankle fusion [68].

Future research should focus on creating a standardized system that combines CT-based fracture patterns, syndesmotic stability, and cartilage damage into clear treatment pathways. Large multicenter studies will help define when and how to fix these fractures, and when extra syndesmotic stabilization is needed. Reporting should not rely on radiography alone, but also include patient-reported outcomes, return-to-work data, and activity levels.

New technologies and materials may also change the way we treat these injuries. Biodegradable screws and suture-button devices can reduce the need for hardware removal. Patient-specific 3D-printed plates and guides, based on CT scans, may allow more precise fixation while reducing soft tissue irritation. Biologic options, such as bone graft substitutes and bioactive coatings, may improve healing in osteoporotic or comminuted fractures [69-71].

Looking further ahead, arthroscopy, intraoperative 3D navigation, and even AI-based imaging could help surgeons achieve more accurate real-time reductions. Rehabilitation should also be part of this progress, with a shift toward earlier mobilization, neuromuscular retraining, and tailored recovery plans that help patients return to work and sports more quickly [72]. In short, the future of PMF management is not just about better fixation; it is about smarter implants, digital technology, and patient-centered care that, together, improve both healing and function.

## Conclusions

The management of PMFs has changed over the years. The critical importance of anatomic reduction has driven the trend toward direct posterior approaches. Success requires careful preoperative planning using CT imaging, appropriate approach selection, meticulous surgical techniques, and structured rehabilitation programs. While small, stable fragments may be managed conservatively in selected patients, the low tolerance for malreduction and high rates of PTOA with suboptimal treatment support appropriate surgical management in most cases. Future research should focus on defining optimal treatment thresholds and developing patient-specific algorithms to improve outcomes.
